# The Mentors in Violence Prevention programme: impact on students’ knowledge and attitudes related to violence, prejudice, and abuse, and willingness to intervene as a bystander in secondary schools in England

**DOI:** 10.1186/s12889-024-18210-9

**Published:** 2024-03-06

**Authors:** Nadia Butler, Zara Quigg, Charley Wilson, Ellie McCoy, Rebecca Bates

**Affiliations:** 1 School of Public and Allied Health, Liverpool John Moores University, Tithebarn Street, Liverpool, L2 2ER UK; 2 School of Nursing and Advanced Practice, Liverpool John Moores University, Tithebarn Street, Liverpool, L2 2ER UK

**Keywords:** Youth violence, Mentors in Violence Prevention, Violence prevention, Bystander intervention, Peer mentoring, Social norms, Secondary school

## Abstract

**Background:**

Violence is a leading cause of death and disability for young people and has serious impacts on prospects across the lifecourse. The education sector is a crucial setting for preventing youth violence through incorporating programmes that address attitudes and behaviours. The Mentors in Violence Prevention (MVP) programme aims to change harmful attitudes and norms, and increase non-violent bystander intervention, through a peer mentoring approach. To date there is limited evidence on the effectiveness of the intervention in UK school settings. The aim of the current study was to evaluate the impact of the programme on students’ attitudes and knowledge related to violence prevention.

**Methods:**

The study employed a mixed methods design. Pre and post surveys measured changes in students’ (aged 11–18) attitudes and knowledge related to violence prevention and bystander behaviour, gender stereotyping, acceptability of violence, and perceptions of others’ willingness to intervene. Interviews/focus groups with programme delivers and students, and anonymised programme data were used to explore and supplement survey findings.

**Results:**

Overall, perceptions of the programme content and delivery were positive. Several beneficial impacts of the programme were found for mentors (students delivering the programme), including significant positive changes on measures of knowledge and attitudes towards violence prevention and the bystander approach, acceptability of violence perpetration, and perceptions of other students’ willingness to intervene (effect sizes were small-medium). However, the study found no significant change on any of the outcomes amongst mentees (younger students receiving the programme from mentors). Despite this, qualitative evidence suggested mentees enjoyed the content of the programme and the peer-led delivery, and this built relationships with older students. Qualitative evidence also identified additional benefits of the programme for mentors, including leadership and communication skills, and increased confidence and supportive relationships.

**Conclusions:**

Evidence from this study suggests MVP is effective as a targeted programme for mentors, but no significant evidence was found to demonstrate its effectiveness as a universal bystander and violence prevention programme for mentees. Whilst further research with more robust study design is needed, developing mentors as leaders in violence prevention is a valuable impact of the programme in its own right.

**Supplementary Information:**

The online version contains supplementary material available at 10.1186/s12889-024-18210-9.

## Background

Globally, an estimated 200,000 homicides occur each year amongst young people aged 10–29 years, making it the fourth leading cause of death for individuals of this age [1]. For every young person who is killed by violence however, many more experience non-fatal forms of youth violence which encompasses physical violence, including weapon carrying; psychological and verbal abuse; gender-based violence (GBV), including sexual violence and harassment; intimate partner violence; and bullying, including cyberbullying [2]. In the United Kingdom (UK), police recorded crime and hospital admissions show that knife crime and assault related injury peaked around 2018/19 [3–5]. Whilst recent years have shown some decrease in incidence, data from 2021/22 showed that one in every six hospital admissions data for assault by a sharp object were amongst young people aged 18 or younger [4]. Youth violence can have serious impacts on young people’s health and wellbeing [6], educational outcomes [7], and social and economic prospects [1, 8], across the lifecourse. Furthermore, experiencing violence during childhood is a risk factor for future violence victimisation [9] and perpetration [10]. Costs associated with youth violence are also high, with recent work estimating that serious youth violence across England and Wales had a total social and economic cost of £11 billion over an eleven-year period [11].

Adolescence is a critical period for the formation of attitudes and beliefs, and in particular for negative or prejudicial attitudes [12]. Individual’s attitudes, beliefs, and behaviours, and group social norms, are key drivers in preventing or perpetuating violence, bullying, and abuse in childhood [13, 14]. Social norms, which are the shared perception about others that exist in a social group, can also influence individual’s motivation to perpetuate, condone, or challenge violence because of the fear of social disapproval, desire to win approval, and the internalisation of perceived normal behaviour [13, 15]. Social influences and social norms are particularly influential during adolescence and play a strong role during this period in the formation of a young person’s attitudes, with this impact shown to be maintained across time into adulthood [16]. Thus, interventions which seek to address negative and prejudicial attitudes, beliefs, and behaviours, in addition to social norms, are key strategies in tackling youth violence and preventing further violence and other adverse impacts across the lifecourse.

As a formative setting for children and young people, the education sector, working in partnership with other stakeholders, including parents and the community, is in a unique position to prevent and address youth violence. Schools have the potential to create an environment where harmful values, attitudes, and behaviours concerning violence, gender, and other prejudice can be changed and principles of equality, tolerance, and respect are promoted and instilled [2]. This can be done through curriculum approaches and teaching methods which educate and equip children and young people with the knowledge and the skills to critically examine prejudicial social norms, engage in healthy peer relationships, manage aggression, develop interpersonal communication and coping mechanisms, and resolve problems in a non-violent way [2, 17]. Incorporating a mentoring model into these types of approaches is an effective way of changing social norms by providing peers with a positive role model who they relate to [18]. The mentoring model is a highly variable approach and there is no consensus as to a single concept of mentoring, with, for example, some programmes using peer mentors, whilst others use adult mentors, and programmes can be on a one-to-one or group basis [19, 20]. The wide spectrum of mentoring models can give rise to highly variable outcomes and levels of effectiveness thus, evaluation of individual programmes is critical. Another important way of changing social norms is incorporating a bystander approach into the curriculum [21–23]. This approach includes learning and practising safe and appropriate skills to identify, speak out against, or seek help from others to respond to incidents of violence or other problematic situations [17]. Bystander approaches aim to move beyond the traditional focus of violence prevention programmes on the victim/perpetrator dichotomy and engage and empower all individuals to play a role in violence prevention thus changing social norms for wider groups than just those at risk of perpetuating or condoning violence [24].

One such programme which uses a peer mentoring model and bystander approach is the Mentors in Violence Prevention (MVP) programme which was first established in the United States in universities and colleges [25], and has since been implemented in secondary school settings across the United States, Scotland, England, and Sweden [26–31]. MVP aims to address harmful attitudes and norms and increase non-violent bystander intervention through a peer mentoring approach to inform and empower individuals to become proactive bystanders in the face of violence and other prejudicial and harmful behaviour [24, 28]. The curriculum includes considering violence through a gendered lens, developing leadership, learning about a bystander approach, understanding the scope of violent behaviour, and challenging victim blaming. The mentors (students delivering the programme) lead their peers, typically from a younger year group (mentees; students receiving the programme) in group discussions of realistic scenarios covering a range of abusive behaviour they might witness as a bystander. A list of several actions which a bystander might consider taking in the situation are then presented and discussed as a group. In line with the original aims of the programme of addressing gender-based and sexual violence, many of the scenarios from the original programme strongly emphasise the importance of gender stereotypes and cultural conceptions of masculinity and femininity in addressing root causes of violence [24, 28]. Adaptation of the programme to other countries and settings has seen the expansion of the programme to cover other forms of violence and abuse by challenging other types of stereotypes and prejudice (e.g., racism, homophobia) [27].

To date MVP has primarily been evaluated in US college populations with empirical studies suggesting evidence for the efficacy of MVP in changing attitudes and norms and preventing GBV and other abusive behaviours [24, 32]. More recently the programme has been implemented in Scotland, with initial qualitative evaluations suggesting it is adaptable to a UK school setting and indicating positive perceptions of MVP in terms of recruitment, training, and implementation processes [26, 33]. However, the quantitative outcome evaluation did not find any significant positive impact of the programme on students, whether as mentors or mentees [34]. MVP is now being delivered across several areas in the United Kingdom, particularly via Home Office funded Violence Reduction Units (VRUs) [35, 36]. Further research is needed to determine if findings from Scotland are replicated across other implementation sites in the UK. Focusing on the delivery of MVP in one VRU area in England (the Merseyside Violence Reduction Partnership [MVRP]), the aim of the current study was to:

1. explore mentors’ and mentees’ perceptions and experience of the programme;
2. identify the impact of the programme on mentors’ and mentees’ knowledge and attitudes related to violence prevention, bystander behaviour, gender stereotypes, and violence perpetration;
3. identify the impact of the programme on social norms related to bystander behaviour; and.
4. explore additional impacts of the programme for mentors and mentees.

## Methods

### The intervention

A detailed description of the MVP programme implemented in the schools included in the current study has been published elsewhere [27, 31] (see also Additional File 1: Figures 1 and 2). Twenty-eight secondary schools across the Merseyside region of the North West of England were recruited to participate in the MVP programme during the 2020/21 and 2021/22 academic years. The schools were selected by the educational lead of MVRP, who were funding the programme. An external youth organisation, Merseyside Youth Association (MYA), specialised in delivery of a range of youth intervention programmes, was commissioned by the MVRP to deliver the programme and these school development officers were trained by a former representative of MVP Scotland. Each school was assigned a school development officer who: conducted the two-day MVP Mentor Support Team Professional Learning Programme with two school staff members who would support programme implementation; delivered the two day training for mentors; provided refresher sessions for mentors before they delivered each session; and supported the supervision of mentors delivery of the sessions to mentees. Each school was asked to commit to the delivery of the two core modules of MVP and select three additional topics (see Additional File 1: Table 1) which they thought were most relevant to their school. Sessions typically took place weekly during a one-hour class period. Not all schools involved in the implementation of MVP took part in all elements of the evaluation.

### Participant recruitment and sample

All programme implementers (*n* = 3), as well as the programme manager (*n* = 1) and MVRP education lead (*n* = 1) were invited to participate an interview. Written consent was obtained from all individuals who agreed to take part.

All twenty-eight schools were approached to participate in the evaluation, of which 14 consented to take part in at least one of the study methods^1^. A passive informed consent process was used to recruit students to the evaluation study. Headteachers or school staff involved in MVP were recruited as gatekeepers and decided on the consent procedure most appropriate for their students, options included parental opt in, parental opt out or gatekeeper loco parentis. Parental opt in consent was the preferred choice and involved parents/guardians receiving an information sheet and consent form prior to commencement of MVP. They were asked to sign and return the consent form if they gave consent for their child to participate in the evaluation. For opt out, parents received a copy of the information and a consent form that they only needed to return if they did not wish for their child to participate in the evaluation. Gatekeepers were asked to provide written consent in loco parentis for children whose parents did not contact them to opt out of the study or if the gatekeeper chose not to send out the opt-out forms. This approach took into consideration the intervention implementers (as gatekeepers) specialist knowledge and experience of working with the young people and COVID-19 procedures which may have restricted communication methods between schools and parents/guardians (e.g., moves to website-based whole school/class communication rather than letters sent home to parents/guardians). Parental consent/gatekeeper loco parentis and student assent was obtained for all participants.

Ethical approval for the study was granted by Liverpool John Moores University Research Ethics Committee (REC no. 21/PHI/006).

### Study design

A mixed methods design was used. This included quantitative measures implemented using pre and post surveys with mentors and mentees. Quantitative data was triangulated with qualitative data from focus groups with mentors, interviews with stakeholders, a school staff survey, and secondary programme monitoring data.

#### Student surveys

Pre (Time 1: before training) and post (Time 2: immediately after training) surveys were implemented with mentors who took part in the training. 426 mentors completed the pre survey and 317 completed the post survey (*N* = 14 schools). Of these, 273 pre and post surveys could be matched (64.1% retention rate) and thus were included in the analysis. The majority of mentors were aged 14–15 years (87.0%; *n* = 127), 2.7% (*n* = 4) were 13, and 10.3% (*n* = 15) were 16–18. Two thirds of the mentors were female (66.9%; *n* = 174). Pre (Time 1: before the first session) and post (Time 2: after the fifth session) surveys were implemented with mentees taking part in the programme. 603 mentees completed the pre survey, and 456 completed the post survey (*N* = 11 schools). Of these, 167 pre and post surveys could be matched (27.7% retention rate) and thus were included in the analysis. The majority of mentees were 12 years of age (91.4%; *n* = 96), and 8.6% (*n* = 9) were aged 11. One in six mentees were female (61.0%; *n* = 97).

Surveys aimed to identify individual level changes in: attitudes related to violence prevention and bystander behaviour; gender stereotyping; violence perpetration; and, social norms related to other student’s willingness to intervene in problematic situations. Surveys included questions on: demographics; perceptions of the training/programme content, delivery and usefulness (post only); and, validated measures including:

***Slaby Bystander Efficacy Scale*** [37]: 8-item scale which measures beliefs about the efficacy of violence prevention. Participants indicate on a five-point scale how much they agree with each item (strongly disagree to strongly agree). Scores on each item are summed to produce an overall total score (range 8–40), with higher scores indicating more positive attitudes towards the efficacy of violence prevention. Cronbach’s alpha was 0.864.

***Bystander Intervention Survey*** [38]: 6-item scale measuring perceptions of leadership skills and attitudes to intervening in problematic situations. Participants indicate on a five-point scale how much they agree with each item (strongly disagree to strongly agree). Scores on each item are summed to produce an overall total score, with higher scores indicating higher better leadership skills and more positive bystander attitudes. Cronbach’s alpha was 0.688.

***Attitudes toward Women scale*** [39]: 12-item scale measuring gender stereotyping. Participants indicate on a five-point scale how much they agree with each item (strongly disagree to strongly agree). Scores on each item are summed to produce an overall total score, with higher scores indicating higher acceptance of gender stereotyping and more negative attitudes towards women. Cronbach’s alpha was 0.845.

***Attitude toward Violence scale*** [40]: 6-item scale measuring attitudes toward violence and its acceptability, particularly in relation to fighting. Participants indicate on a five-point scale how much they agree with each item (strongly disagree to strongly agree). Scores on each item are summed to produce an overall total score, with higher scores indicating higher acceptance of violence and limited use of nonviolent strategies. Cronbach’s alpha was 0.746.

***Generalized Perception of Peers scale (adapted)*** [41]: 4-item scale adapted from the original scale [42] measuring informal social control through assessing perceptions of other student’s frequency of intervening in problematic situations. Participants indicate on a four-point scale how often they think other students would intervene (never to always). Scores on each item are summed to produce an overall total score, with higher scores indicating perceived higher frequency of other student’s likelihood to intervene. Cronbach’s alpha was 0.916.

***Student Resilience Survey*** [43]: is comprised of 11 subscales which measure different sources of resilience. The current study used the participation in school life subscale (comprised of 2 items) to measure perceptions of connectedness to school. Participants indicate on a five-point scale how often they feel connected to their school (never to always). Scores on each item are summed to produce an overall total score, with higher scores indicating higher participation in school activities. Cronbach’s alpha was 0.656.

#### Staff survey

An online survey was distributed to school staff who had taken part in the MVP training. 19 staff (*N* = 10 schools) completed the survey. The survey explored staff perceptions on the MVP training, programme implementation in their school, sustainability, facilitating factors and challenges or barriers to implementation, and areas for development.

#### Focus groups, interviews, and programme monitoring data

Two focus groups were conducted with mentors (*n* = 15) trained in MVP and involved in programme implementation. Focus groups took approximately 30 min and were carried out in person. Qualitative semi-structured interviews were conducted with stakeholders (*n* = 6) who had a key role in the implementation of the intervention, this included the external programme team, school staff, and commissioners of the programme. Interview length ranged from 43 min to one hour 32 min and were carried out online. Interviews and focus groups explored: perceptions of the training; perceptions of delivering the programme; factors supporting and impeding implementation of MVP; areas for development; and perceived impacts on mentors, mentees, school staff and the wider school.

The post mentor and mentee surveys, and the staff survey included free text questions. The MYA external programme delivery team also informally captured qualitative feedback on perceptions and impacts of the programme from mentors, mentees, and school staff, through a range of methods including film, discussion groups, graduation ceremonies, and feedback sheets. Qualitative survey responses and secondary feedback data were analysed alongside the interview and focus group data.

### Analyses

Quantitative analyses were undertaken in SPSS (v.27). Paired sample t-tests were used to examine changes in the six measures from pre to post survey and post-hoc tests calculated the effect sizes, with Cohen’s categorisation of effect sizes (small, 0.10; medium, 0.30; large, 0.50) used to determine the magnitude of effect. The Bonferroni Correction method was performed to account for running multiple comparisons on each scale. Focus groups and interviews were audio recorded and transcribed verbatim. Transcripts, secondary feedback data, and staff and student survey responses to qualitative questions were imported into NVivo Enterprise and thematically analysed [44]. Themes were generated through the use of inductive and deductive analysis, specifically using deductive analysis to support or refute quantitative analysis and inductive analysis to explore potential additional impacts not measured through quantitative measures. For each quote a brief descriptor is given indicating participant role (mentor, mentee, school staff, stakeholder) and data source (I, interview; FG, focus group; SD, secondary programme monitoring data; S, student survey; SS school staff survey). We applied triangulation in the analysis and interpretation of the findings, with qualitative and quantitative results presented and arranged together to reflect and describe perceptions and impact of the programme.

## Results

### Perceptions of the MVP programme

Qualitative findings suggested that overall perceptions and experiences of the MVP programme were positive from mentors, mentees, and school staff. Several themes were identified related to the content and delivery style of the programme which influenced these perceptions.

A key premise of MVP is the peer delivery approach and qualitative findings suggested both mentees and mentors felt this is what worked best about the programme. Mentees reported finding it easier to understand and discuss the topics because they were delivered by a fellow student rather than a teacher and this was also observed by school staff.

“*Our younger students responded really well to being led by older students as opposed to teachers and this resulted in meaningful discussions which then led to small, but relevant and positive cultural and attitudinal shifts with the year group.”* (School staff, SS).

Mentors perceived mentees as more likely to listen to them because of shared perspectives and experiences. Both mentors and mentees discussed the safe space that was created between students to discuss topics and voice opinions which they may not have shared with a teacher.

*“We are telling them from our perspective, and because we are older, we may have seen it. Like I said, they might listen to us more than a teacher.”* (Mentor, FG).

*“I enjoyed exploring gendered violence. I had written down a list of the pressures I felt that often overwhelmed me. It was informative and I was able to express myself and my fears without being labelled a ‘man hater’.”* (Mentor, S).

Mentors and mentees generally had a positive perception of the topics covered and resources used. Some mentees felt there needed to be more focus on gender, and GBV along with a deeper discussion of some topics. It was also noted by mentees that prior warning was needed about the sensitive nature of the topics about to be discussed.

*“I think the whole programme was useful as it showed real stuff that could happen and how to prevent it.”* (Mentee, S).

*“I really enjoyed learning about the statistics and factual information on violence because it makes pupils realise what is going on in our world and how we can prevent it.”* (Mentor, S).

The optional three topics which each school includes in the programme is decided using a survey (implemented by the MYA delivery team) prior to programme implementation where staff and students can identify the issues most relevant to their school from a list of potential topics. Content of the MVP programme was also adapted to use local examples and statistics relevant to the local community. School staff reported that students’ ownership over the content of the optional sessions was important and *“helped to facilitate their positive engagement with the programme.”* (I).

*“It’s really highlighted the importance of youth voice and asking them, what are the issues that you see on the corridor and what it allows us to do as an organisation is tailor that MVP curriculum to try and combat those very real issues that are happening.”* (Stakeholder, SD).

*“It’s the shock factor, I think it sort of gives the young people that buy in. This isn’t just something that’s happening in London, and isn’t my business, on my doorstep… it really highlights the need for a programme like MVP. So we’re talking about knife crime, but we’re talking about it in Liverpool City Centre, we’re talking about it right, by your school, right by your home. And I think it brings the programme alive in a way and obviously young people, I think, to want to do more about it.”* (Stakeholder, I).

The interactive nature of MVP programme delivery was also highlighted as being key to supporting engagement and interest with the topics. Mentors noted how MVP provides an opportunity to discuss topics that are only discussed during PSHE lessons (statutory UK requirements for schools to cover topics such as internet safety and harms, relationships, pornography, violence against women and girls) but in a more interactive and engaging way.

*“I think MVP is needed in schools because the only time we get to talk about stuff like this other than MVP is in PSHE… we learn lots but then it’s more like on paper and it’s less in person in a way.”* (Mentor, FG)

*“It was a different way we could get more, often difficult social and emotional conversations to happen and it allowed the pupils to engage in a different format, so where they aren’t being spoken to or lectured. They weren’t just sitting there writing they were having peer conversations, and everyone was focused on the same thing.”* (School staff, I).

Mentors reported that sessions could be made even more interactive as they felt mentees became distracted and bored during parts when they were delivering large amounts of information, and mentees echoed this suggesting more interaction between students.

*“More fun activities where we get to interact with each other and interact with the mentors.”* (Mentee, S).

*“I think something that worked really well were some activities like the agree, disagree, unsure and other things, these really got the children involved and felt more inclined to participate.”* (Mentor, S).

**Table 1:** Changes in quantitative outcome measures, mentors and mentees

	Mentors							Mentees						
Item	*n*	Time 1 Mean (SD)	Time 2 Mean (SD)	Mean change (95% CIs)	t	*p*	d	n	Time 1 Mean (SD)	Time 2 Mean (SD)	Mean change (95% CIs)	t	*p*	d
Attitudes towards efficacy of violence prevention total score^1^	138	32.77 (3.327)	34.29 (5.616)	-1.52 (-0.66 - -2.38)	-3.489	< 0.001^	-0.30	70	31.50 (4.323)	29.51 (6.788)	1.99 (0.41–3.56)	2.519	< 0.05	0.30
Bystander attitudes total score^2^	240	22.24 (2.815)	23.23 (3.660)	-0.99 (-0.54 - -1.43)	-4.348	< 0.001^	-0.28	108	18.98 (3.329)	18.71 (3.724)	0.27 (-0.41-0.95)	0.779	NS	0.08
Perceptions of other students’ bystander behaviour total score^3^	103	9.21 (2.796)	10.69 (2.737)	1.48 (0.87–2.08)	-4.868	< 0.001^	-0.48	37	11.89 (4.047)	11.70 (2.837)	0.19 (-1.12-1.49)	0.294	NS	0.05
Attitudes towards violence total score^4^	82	15.62 (3.946)	14.35 (4.129)	-1.27 (0.63–1.91)	3.935	< 0.001^	0.44	52	17.94 (5.493)	18.19 (5.080)	-0.25 (-1.98-1.48)	-0.290	NS	-0.04
Gender stereotyping total score^5^	104	20.30 (5.953)	20.40 (6.840)	-0.11 (-1.00-0.792)	-0.234	NS	-0.02	36	20.61 (5.520)	22.03 (7.952)	-1.42 (-3.50-0.67)	-1.377	NS	-0.23
School participation total score^6^	115	5.68 (1.852)	6.11 (1.914)	-0.44 (0.16 - -0.76)	-2.691	< 0.01^	-0.25	60	5.18 (1.987)	4.85 (2.138)	0.33 (-0.16-0.82)	1.364	NS	0.18

Notes. ^**1**^Higher score indicates more positive attitudes towards the efficacy of violence prevention. ^**2**^Higher score indicates better leadership skills and more positive bystander attitudes. ^3^Higher score indicates perceived higher frequency of other student’s likelihood to intervene. ^4^Higher score indicates higher acceptance of violence and limited use of nonviolent strategies. ^**5**^Higher score indicates higher acceptance of gender stereotyping and more negative attitudes towards women. ^6^Higher score indicates higher participation in school activities. SD, standard deviation. ^Statistically significant when controlling for multiple testing (Bonferroni correction)

### Violence prevention and bystander knowledge and attitudes

There was a statistically significant increase in mentors’ total score on the efficacy of violence prevention measure from Time 1 to Time 2, with a mean increase in scores of 1.52 (Table 1). The eta squared statistic (0.30) indicated a small effect size. There was a statistically significant decrease in mentees’ total score on the efficacy of violence prevention measure from Time 1 to Time 2, with a mean decrease in score of 1.99 (Table 1). The eta squared statistic (0.30) indicated a small effect size. There was a statistically significant increase in mentors’ total score on the bystander attitudes measure from Time 1 to Time 2, with a mean increase in scores of 0.99 (Table 1). The eta squared statistic (0.28) indicated a small effect size. There was no significant change in mentees’ total score on the bystander attitudes measure from Time 1 to Time 2 (Table 1).

Qualitative data supported these findings with mentors reporting increased knowledge related to violence, feeling better equipped to recognise risks and warning signs, and knowing how to intervene. Mentors also provided examples of situations where they had practiced bystander intervention behaviour. There was less evidence from mentees about the impact of the programme on their knowledge and attitudes.

*“MVP teaches you life skills on mental health and violence. It enlightens you on the effects that cause and prevent violence. For example, gender lenses, victim blaming, bystanding, abuse, violence, and leadership. We have learned how to show these skills during our learning. Overall, we are confident in showing people what leads up to violent actions and what changes we can make to stop them. We are Mentors in Violence Prevention.”* (Mentor, SD).

*“I have heard some of the pupils who are being mentored talk about the MVP programme and what they have learnt on the yard at lunch time.”* (School staff, S).

*“Made me aware of what’s going on, if something’s going wrong or someone is mistreating someone in the group, I now know about the bystander approach, and I’d say something now.”* (Mentor, SD).

*“I would have walked away from a fight before MVP but now I walk away and go and tell a teacher.”* (Mentor, SD).

*“I told them to stop and walk away. Asked the kid if they were alright, then reported the problem.”* (Mentor, S).

*“Thinking about what we would do in a bad situation involving bullying or rumours being spread.”* (Mentee, S).

### Perceptions of other students’ bystander behaviour

There was a statistically significant increase in mentors’ total score on the perceptions of other students’ bystander behaviour measure from Time 1 to Time 2, with a mean increase in scores of 1.48 (Table 1). The eta squared statistic (0.48) indicated a medium effect size. There was no significant change in mentees’ total score on the perceptions of other students’ bystander behaviour measure from Time 1 to Time 2 (Table 1).

### Attitudes towards violence perpetration

There was a statistically significant decrease in mentors’ total score on the attitudes towards violence measure from Time 1 to Time 2, with a mean decrease in scores of 1.27 (Table 1). The eta squared statistic (0.44) indicated a medium effect size. There was no significant change in mentees’ total score on the attitudes towards violence measure from Time 1 to Time 2 (Table 1).

### Gender stereotyping

There was no significant change in mentors’ or mentees’ total score on the gender stereotyping measure from Time 1 to Time 2 (Table 1).

### School participation

There was a statistically significant increase in mentors’ total score on the school participation measure from Time 1 to Time 2, with a mean increase in scores of 0.44 (Table 1). The eta squared statistic (0.25) indicated a small effect size. There was no significant change in mentees’ total score on the school participation measure from Time 1 to Time 2 (Table 1).

School staff reported that mentors were usually selected from a group who weren’t necessarily the high achieving students, or those involved in extra-curricular activities, and could often include students who were having difficulties with behaviour or academic work. School staff gave several examples of the positive impact MVP involvement had for these mentors. One staff member reported how the programme supported better communication and engagement with the parents of a mentor who was previously having problems.

*“Pupils and their families know they are thought highly of in school. One student delivering the programme is on report for behaviour. Usually parents are not supportive, but since she has been leading on the MVP programme, parents are supporting the school.”* (School staff, I).

### Relationships

Development of supportive adult and peer relationships was recognised by most participants as a major impact of the programme for mentors and mentees. School staff reported that mentors supported each other during the delivery of the session and worked well as a team, whilst mentors reported making new friends with other mentors.

*“Mentors supported each other prior to delivering by talking to each other and encouraging each other. Some mentors took over bits that other people missed out; they worked as a team brilliantly. Beforehand, they tried to play to their own strengths, but on the day, you have to adapt yourself and for the class in front of you.”* (School staff, I)

*“It was easy for me to get involved as it took my mind off everything. The programme was good, the best thing about it was working in a group with people that I don’t usually hang around with.”* (Mentor, SD).

Relationships between the mentors and mentees were also seen as an important factor in the success of MVP and in supporting increased bystander behaviour. MVP brought mentors and mentees together to meet new people and mix with young people they would not normally engage with. This was seen as a good way to develop social networks and social skills. Mentees recognised the mentors as good role models. This was viewed as particularly important in supporting mentees to adopt behaviours demonstrated by mentors and build resilience, but also in terms of mentors having pride in their role and recognising their achievements within the programme. Stakeholders acknowledged the importance of having positive role models in society and within local communities to support young people’s aspirations and to provide young people with a trusted person to turn to when needed. This was also seen as a positive way to encourage bystander intervention behaviour and reporting of violence.

*“The MVP programme means a lot because it gives us like a personal relationship with the younger years and I think it is important to have like key relationships with the younger years. Especially like whether everything that’s going on, all the issues that arise, I think I firmly believe that we do create a safe space for the kids.”* (Mentor, SD).

*“The MVP programme has been completely such an amazing opportunity and one that I never thought I’d be able to experience, and one that I think is quite once in a lifetime. It’s been so great to socialise with people I wouldn’t normally talk to and teach them about things that I would normally not speak to anyone about.”* (Mentor, SD).

*“I think there is possibly a bigger impact as the younger ones listen to their peers and then that relationship also develops over the long term.”* (School staff, SD).

*“Enjoyed having year 10 people in charge because they know what it is like to be a student.”* (Mentee, S).

*“Because it’s like, although still kids ourselves and we’re still in school… If they didn’t want to go to an adult, they want to go to someone more their age, we’ll go to him because he knows what he’s doing. He knows what he’s talking about.”* (Mentor, FG).

The relationships established between the programme implementers and mentors during the programme were seen as key in supporting the mentors to build skills and develop confidence. The close working relationships meant that the mentors felt supported in their role and felt part of the development and delivery of MVP and valued throughout. It was clear from mentor feedback that a key part of the enjoyment of the programme was working with the programme implementors with many mentors mentioning them by name and reporting how much they enjoyed their approach. Mentors reported feeling able to voice their opinions to the external programme implementers more easily than to teachers, where they might fear disapproval.

*“Input from [programme implementor] was fantastic. Students formed really positive relationships with them during the training which resulted in them communicating with them afterwards when seeking advice and support with aspects of the programme.”* (School staff, S).

*“I loved how it was a safe space for everyone to express their opinions. You were never told your opinion was wrong or invalid but [encouraged] to broaden your view.”* (Mentor, SD).

### Leadership, confidence, and communication skills

Staff and mentors reported improvements in communication, presenting, teaching, improvisation skills, computer skills, public speaking, and time keeping. Further mentors reported how they learnt to recognise when mentees were not understanding the programme content and adapted their delivery accordingly.

*“I’m definitely a lot better at communication where before I may not have been ok at continuing a conversation”.* (Mentor, SD)

*“I think I’ve definitely learned to adapt to the needs and like the preferences of the different children because all groups of children are, it’s a wide variety, so some might suffer with learning disabilities and also might be anxious and things like that, so learning to overcome that. I’m making it a comfortable place with them and making it a space where they are OK with talking to others.”* (Mentor, SD).

Mentors reported increased confidence and self-esteem, recognised by stakeholders as important skills and necessary for bystander intervention behaviour. Both staff and mentors reported impacts for mentors in terms of leadership skills and perceiving themselves as a role model to others.

*“Greater confidence, maturity, and empathy. Mentors felt empowered to speak to young students and deliver some challenging topics. They have developed their communication skills and have discovered that their message has been listened to. After their final session of the year many of the mentors commented on how much they had enjoyed the experience and that they were amazed by the fact that they were capable of being mentors to the younger students.”* (School staff, S).

*“Because of MVP I’ve become a lot more confident when speaking because it’s kind of forced myself to take on more of like a hands-on role. So, we’ve done assemblies not just to like the younger students, but also to our year group in year 11, and I don’t think I’d normally have the confidence to be able to do that. So having MVP has been a really amazing opportunity to grow in confidence.”* (Mentor, SD).

*“The development of the mentors as leaders within the school has been a huge success. We chose a group of rather ‘untypical’ students and it has been a delight to see them grow in confidence when delivering the sessions.”* (School staff, S).

*“I think one of the like main benefits of MVP is that obviously you gain lots of leadership skills and being involved with younger pupils and getting to have involvement with their lives at your school.”* (Mentor, SD).

*“The school has ‘’Leadership’’ in its name, and this programme and what you have done has done exactly that. It’s been remarkable… I’m not quite sure I’ve seen any projects like MVP in 30 years of teaching, it’s amazing.”* (School staff, SD).

Staff and mentors spoke about the potential impact gaining or improving all these types of skills would have on mentors beyond just delivery of the MVP programme, and that they were particularly relevant to other schoolwork and future employment.

*“One skill that I left out was to teach children. I’d never really had the opportunity to do that before, so it was really nice and to see what they responded with and how they interact. I think that was really useful, I could definitely use that later on in life.”* (Mentor, SD).

*“The MVP sessions this morning was amazing again, they’re really coming into their own! [Mentor] was complimented on his ability and that he could be a future teacher!”* (School staff, SD).

*“I’m thinking specifically of one mentor. And he had difficulties and he was selected. And I think it was more about the head teacher said you know what this guy would be coming out of school with very little as far as academic ability, what I’m seeing from here is that, you know, he has personified what this school is all about. We’re training people up to be leaders of which he is.”* (Stakeholder, I).

## Discussion

Youth violence is a serious public health problem globally with high individual and societal costs [6, 7, 9, 11]. In recent years it has received increased attention in the UK, with a focus on preventing and responding to youth violence taking a public health approach, including universal and targeted violence prevention programmes implemented in educational settings, such as the Mentors in Violence Prevention (MVP) programme [26]. Evidence from implementation of the programme in the United States suggests MVP is effective in addressing harmful attitudes and norms, and increasing non-violent bystander intervention, through a peer mentoring approach [24, 32]. However, to date there is limited evidence on the acceptability and effectiveness of the intervention when translated to UK school settings. Overall, the current study found that school staff, mentor, and mentee perceptions of the programme content and delivery were positive, and the programme had a number of positive impacts for mentors in particular.

The original premise of the MVP programme focuses on the gendered nature of violence and associated sexist attitudes, and the overarching aim was to reduce social acceptability of such behaviours using peer leaders [24]. Previous evaluations of MVP in the US have demonstrated positive changes in awareness and knowledge of GBV, and attitudes towards women [28, 45]. However, the current study found no significant impact of the programme on the measure of gender stereotyping for mentors or mentees. A high ceiling effect at baseline may partly explain the lack of significant change, however the adapted content of the programme in the current study site may have reduced the focus and thus impact of the programme on gender stereotypes. Whilst the gendered nature of violence is included as one core session in Merseyside’s implementation of the MVP programme, the focus on GBV may have been diluted at the expense of including other optional topics that are not necessarily associated with sexist attitudes or considered GBV (e.g. racism, county lines, insults, online abuse). Evaluations of MVP in Scotland [26, 34, 46], Sweden [29], and the West Midlands [30], have also reported little impact of the programme on gender stereotypes and sexist attitudes. Observations of sessions in Sweden demonstrated that whilst some activities such as the gender box, were reported by students as a fun exercise to discuss what is specific to boys and girls, there was less discussion about how to challenge gender norms [29]. Similarly in the current study, whilst mentors and mentees reported enjoying the gender aspect, some students reported that there needed to be a stronger focus on it. It is possible that an increased focus on gender stereotypes, and crucially how to challenge stereotypes and negative attitudes, would increase the impact of MVP on harmful attitudes associated with GBV.

Whilst Katz [24] warns against shifting the focus of MVP away from GBV, the broader scope of Merseyside’s MVP programme had the potential to positively impact attitudes towards violence and bystander intervention more broadly. Such impacts have previously been argued to be beneficial even within primarily GBV focused bystander interventions [47, 48]. The current study found a significant positive impact of MVP on mentors’ attitudes related to violence more generally, indicating reduced acceptability and justification for the use of violent strategies to resolve confrontational or challenging circumstances. Previous research has shown that attitudes towards violence and use of violence are positively associated amongst adolescents, and a meta-analysis concluded modifying attitudes is an important aim for school-based violence prevention programmes [49]. Crucially, in addition to reducing mentors’ attitudes to perpetrating violence themselves, findings demonstrated the programme had a positive impact on mentors’ attitudes related to the efficacy of violence prevention and their willingness to intervene as a witness to violence and abuse perpetrated by others. Previous research has highlighted the importance of personal relevance in encouraging engagement with violence prevention education and activism [50, 51]. Qualitative findings from the current evaluation emphasised how important the locally adapted content of the programme and relevancy of topics was to facilitating engagement and impact of the programme. GBV is a core session of MVP which all students complete, however optional topic sessions are decided by the students and the staff based on which issues are most relevant to their individual school. Further research is needed, but it may be that current findings of positive changes in attitudes towards violence more generally, but not in GBV attitudes, are related to perceived personal relevancy of topics.

Despite the positive changes observed in mentors’ attitudes, no significant quantifiable impact of the programme was observed for mentees. This is in line with previous evaluations in Scotland and the West Midlands [30, 34, 46]. Two key drivers in bystander behaviour are perceptions of others’ willingness and likeliness to intervene, and leadership skills. In the current study, mentors, but not mentees, perceived other students as more willing to intervene following involvement in the programme. Studies have shown that perceptions of other’s willingness to intervene to prevent violence is the strongest predictor of individual’s own willingness to intervene, accounting for up to 42% of the variance in individual’s willingness to do so [52]. This is because social norms related to how to behave are perpetuated not just by what individuals believe to be right or true but also by what they perceive as others in their social group believe is right of true [53]. Furthermore, if social norms and individual attitudes are incongruous, social norms have been demonstrated to be a bigger driver of behaviour [54, 55]. Mentors have more opportunity in their smaller group to discuss attitudes with other mentors and observe how individuals’ attitudes change, however for the larger group of mentees observing any changes in their fellow peers’ attitudes is more difficult and this therefore may explain the differential outcome.

Both qualitative and quantitative evidence from the current study demonstrated mentors, but not mentees, were more likely to perceive themselves as leaders following involvement in the programme. Evaluation of the programme in Scotland also found that mentors, but not mentees, gained leadership skills. The original developer of the programme, Katz remarked “being an active bystander requires someone to possess the qualities of a leader precisely because it is not easy for men—or women—to intervene and challenge abusive behaviours or the belief systems that foster the conditions within which they occur” [24]. Katz argued that that MVP is better described as a leadership programme rather than a bystander programme, because in order to be an effective bystander an individual has to assess a situation, consider their options and take action; factors which are basic leadership protocol [24]. Whilst fulfilling the role of a mentor may develop leadership skills, mentors may represent a group who already have some of these traits. Mentors are usually invited to volunteer for the role or apply to be a mentor and are selected on the basis of having social standing and the potential to be a role model to their peers [56]. Critically, the current study found mentors had a higher mean baseline score on items related to leadership, compared to mentees. Similarly, a US study found the programme was especially effective with individuals who were already invested in the idea of becoming better leaders, for example amongst sports teams and military units [24]. Mentees lower baseline score on leadership skills and the limited opportunity to develop leadership skills compared to mentors may therefore explain the differential impact of the programme on measures of bystander attitudes and knowledge.

Further research is required, but the pedagogy of the MVP programme may be a critical mechanism of change and may explain the differential impact on mentors and mentees. Pedagogy refers to the way curriculum is delivered and can include a variety of methods which increase young people’s engagement with educational content and support them to learn more effectively [17]. The signature pedagogy of the MVP programme is its use of open, lively, and sometimes contentious interactive group discussions [24]. This contributes to a learner centred pedagogy approach which puts the mentors and mentees at the centre of the teaching and learning process, as opposed to teacher-centred approaches where they would passively receive information from teachers. The pedagogy of MVP prioritises dialogue and group process over the rote imparting of information [24], and mentors arguably have greater exposure to this in their smaller group, than mentees who are part of a larger group being led in discussions by mentors in circumstances that more closely resemble traditional class-based learning and thus have less opportunity to practice and fulfil leadership roles. Several mentees in the current study commented on the need for sessions to be more interactive, suggesting they did not have the same opportunity as mentors for engaging in group dialogue. This was reinforced by mentors who felt at times mentees became bored, particularly in parts where there were long sections of information. Findings from the process evaluation of the programme in Scotland recommended mentees should be in groups of no more than fifteen to adequately facilitate discussion and engagement [26]. Thus, future programme implementation should consider reducing the mentee group size and increasing engagement in the sessions, similar to mentors’ experience, to maximise the potential impact of the programme for mentees.

Whilst the key premise of MVP is the peer mentoring delivery model, the differential impact of the programme may also relate to mentors having the programme delivered to them by adults compared to mentees being led in the discussions by fellow peers. Thus, the fidelity and quality of programme delivery may have differed between the two groups, with the experienced external youth workers better able to effectively communicate the messages and engage young people. However, further research is required as evidence suggests that peer-led and adult-led school-based programmes are equally effective [57]. Another key component of MVP was it provided the space to voice opinions without fear of disapproval from a teacher, this was particularly the case for mentors who were led in discussions by the external provider. Similarly, mentees reported being able to express their opinions to their peer mentors which they would have been unwilling to discuss with teachers. However, unlike delivery to mentors, mentor to mentee delivery is usually supervised by teachers and this may have been a barrier to engagement in the discussions. Future research should explore whether a mixed model approach of non-teaching staff and peers improves the impact of the programme on mentees. Whilst the delivery of the programme may have been a factor, the close relationships developed between the mentors and the external MVP programme team, which was cited as a key positive of the programme, may also have been a key mechanism of change for the mentors. Previous research has found that non-parental adult supportive relationships are important factors in promoting and protecting wellbeing and resilience [58], and psychosocial functioning including self-esteem, behaviour, and attitudes towards school [59]. This is the case for all children, but in particular for those who may be experiencing adversity or other difficulties [58]. As previously discussed, mentors are usually chosen because they represent a more vulnerable group who are typically not as engaged in school or extra-curricular activities and who may have had previous conduct and behavioural issues in school. Critically, mentors demonstrated increased feelings of school participation following engagement in the training with the providers. This contrasted with mentees’ experience who did not have the opportunity to develop a supportive adult relationship with the external MVP programme team and was reflected in the lack of positive change in feelings of school participation for mentees.

Despite the positive changes for mentors, many of the effect sizes were small and for mentees there was generally no significant impact. These findings are in line with other evaluations of MVP elsewhere, and with other similar violence prevention programmes (e.g. Street Doctors) [34, 60]. Whilst this may raise a question as to the effectiveness of such programmes, recent work has explored the relevance of Cohen’s conventional effect size cutoffs (small, 0.2; medium, 0.5; and large, 0.8) for school-based interventions and critically, suggests that the conventional thresholds overestimate expected effect sizes in the real-world context of school-based violence prevention programmes [61]. Whilst this may be the case, it is crucial to continue to develop the evidence base by utilising more robust evaluation designs. For example, the current study’s design (pre/post), similar to many other violence prevention programme evaluations, meant there was a lack of an appropriate control group which limits the ability to establish the programme as the casual mechanism of change. The small sample size for each of the paired t-tests may also have contributed to the relatively small effects sizes, and future research should ensure the sample size is large enough so that the analysis is sufficiently powered. We also could not control for between school differences in delivery or fidelity to the programme because numbers were too small to account for this. Future research should therefore include such data on delivery (e.g. year groups chosen as mentors/mentees, gender make up of groups), adaptations to programme content, and school level factors (e.g. deprivation) in analyses to explore potential moderating factors on outcomes. Moreover, not all schools who implemented MVP took part in the evaluation and there may be differences between schools who were willing to take part in implementation and evaluation, those who took part in implementation only, and schools who did not participate in the programme. Further research using more rigorous evaluation designs, such as a randomised control trial, are required to test specific hypotheses based on the findings in this paper.

## Conclusion

To our knowledge this is the first UK evaluation of MVP to find significant positive quantifiable impacts of the programme on students. Whilst more robust evaluation designs are needed, findings from the current study highlighted the positive impact of the programme on mentors’ attitudes towards violence, violence preventability, and bystander approach, and perceptions of other students’ willingness to intervene, in addition to an increase in leadership skills, confidence, supportive relationships, and school connection. However, the anticipated impact of the programme focused on mentees as the primary programme beneficiaries, and whist qualitative findings showed that mentees enjoyed the concept of the programme, no positive quantifiable changes in programme aimed outcomes were observed. This suggests that MVP, at least in the format which it was implemented and evaluated in the current study, may be a more effective targeted rather than universal violence prevention programme. Despite this, there is potential to adapt the programme to ensure the experience of mentees more closely mirrors that of the mentors and thus has the potential to bring about a similar impact. Regardless, developing mentors as leaders in violence prevention and the bystander approach is a critical means of preventing and responding to youth violence and should be considered a valuable impact of the programme in its own right. As one mentor aptly put:

MVP teaches you life skills on mental health and violence. It enlightens you on the effects that cause and prevent violence. For example, gender lenses, victim blaming, bystanding, abuse, violence, and leadership. We have learned how to show these skills during our learning. Overall, we are confident in showing people what leads up to violent actions and what changes we can make to stop them. We are mentors in violence prevention.

### Electronic supplementary material

Below is the link to the electronic supplementary material.

Supplementary Material 1

Supplementary Material 2

## Data Availability

The data generated and analysed in the current study is available from the corresponding author on reasonable request.

## Abbreviations

MVP: Mentors in Violence Prevention
MVRP: Merseyside Violence Reduction Partnership
MYA: Merseyside Youth Association
UK: United Kingdom

## Footnotes

1 Schools which elected not to take part in the evaluation did so primarily because of resource issues (i.e. were not able to provide the time to implement surveys).

## References

[1] Organization WH. Youth Violence, World Health Organization, 8 June 2020. [Online]. Available: https://www.who.int/news-room/fact-sheets/detail/youth-violence. [Accessed 16 Feb 2023].

[2] UNESCO (2017). School violence and bullying: global status report.

[3] Digital NHS. Hospital admissions for assault by sharp object, NHS Digital, 17 June 2019. [Online]. Available: https://digital.nhs.uk/data-and-information/find-data-and-publications/supplementary-information/2019-supplementary-information-files/hospital-admissions-for-assault-by-sharp-object. [Accessed 07 Feb 2023].

[4] Allen G, Burton M (2023). Knife crime in England and Wales: statistics.

[5] Ministry of Justice., Knife and offensive weapon sentencing quarterly, England and Wales, 16 January 2020. [Online]. Available: https://www.gov.uk/government/statistics/knife-and-offensive-weapon-sentencing-statistics-july-to-september-2019. [Accessed 07 Feb 2023].

[6] Schlack R, Ravens-Sieberer U, Petermann F (2013). Psychological problems, protective factors and health-related quality of life in youth affected by violence: the burden of the multiply victimised. J Adolesc.

[7] Wilczak AR. The relationship between youth violence, victimization, and educational outcomes. Sociology Mind. 2014;4:4.

[8] Zielinski DS (2009). Child maltreatment and adult socioeconomic well-being. Child Abuse Negl.

[9] Butler N, Quigg Z, Bellis MA. Cycles of violence in England and Wales: the contribution of childhood abuse to risk of violence revictimisation in adulthood. BMC Medicine. 2020;18:325. 10.1186/s12916-020-01788-3 PMC7667802 33190642

[10] Ttofi MM, Farrington DP, Losel F (2012). School bullying as a predictor of violence later in life: a systematic review and meta-analysis of prosepctive longitudinal studies. Aggress Violent Beh.

[11] Irwin-Rogers K, Muthoo A, Billingham L (2020). Youth Violence Commission final report.

[12] Kinder DM, Sanders LM (1981). Prejudice and politics: symbolic racism versus racial threats to the good life. J Personal Soc Psychol.

[13] World Health Organization (2019). School-based violence prevention: a practical handbook.

[14] Lilleston PS, Goldman L, Verma RK, McCleary-Sills J (2017). Understanding social norms and violence in childhood: theoretical underpinnings and strategies for intervention. Psychol Health Med.

[15] Bandura A. Social cognitive theory for personal and social change by enabling media, in *Entertainment-education and social change: History, research, and practive*, Mahwah, NJ, Lawrence Erlbaum, 2004, pp. 75–96.

[16] Hjerm M, Eger MA, Danell R. Peer attitudes and the development of prejudice in adolescence. SOCIUS: Sociological Reseach for a Dynamic World. 2018;4:1–11.

[17] Women UN, UNESCO and (2016). Global guidance: school-related gender-based violence.

[18] Mentoring, Foundation B (2010). Peer mentoring in schools.

[19] Wood S, Mayo-Wilson E (2012). School-based mentoring for adolescents: a systematic review and meta-analysis. Res Social Work Pract.

[20] Weinberger SG (2005). Developing a mentoring program. Handbook of Youth Mentoring.

[21] Karna A, Voeten M, Little TD, Poskiparta E, Kaljonen A, Salmivalli C (2011). A large-scale evaluation of the KiVa antibullying program: grades 4–6. Child Dev.

[22] Palmer SB, Abbott N (2017). Bystander responses to bias-based bullying in schools: a developmental intergroup approach. Child Dev Perspect.

[23] Coker AL, Bush HM, Brantaco CJ, Clear ER, Recktenwald EA (2019). Bystander program effectiveness to reduce violence acceptance: RCT in high schools. J Family Violence.

[24] Katz J. Bystander training as leadership training: notes on the origins, philosophy, and pedagogy of the mentors in violence prevention model. Violence Against Wom. 2018:1–22. 10.1177/1077801217753322 29542404

[25] Katz J (1995). Reconstructing masculinity in the locker room: the mentors in violence prevention project. Harv Educational Rev.

[26] Williams DJ, Neville FG (2017). Qualitative evaluation of the Mentors in Violence Prevention pilot in Scottish high schools. Psychol Violence.

[27] Butler N, Bates R, Quigg Z (2021). Evaluation of the mentors in Violence Prevention (MVP) Programme across Merseyside - Final Report.

[28] Katz J, Heisterkamp A, Fleming M (2011). The social justice roots of the mentors in violence Prevention Model and its application in a high school setting. Violence against Women.

[29] Bruno L, Joelsson T, Franzen AG, Gottzen L (2020). Heroes and others: tensions and challenges in implementing mentors in Violence Prevention in Swedish schools. J Gender-Based Violence.

[30] Fox C, Ralph N, Harrison E, McElwee J, Bagnall C, Garnett N (2020). Evaluation of the Mentors in Violence Prevention (MVP) programme in the West Midlands.

[31] Butler N, Wilson C, Bates R, Quigg Z (2022). Evaluation of the mentors in Violence Prevention (MVP) programmes across Merseyside 2021/22.

[32] Katz J, Moore J (2013). Bystander education training for campus sexual assault prevention: an intitial meta-analysis. Violence Vict.

[33] Scotland E. MVP Scotland: Progress report 2018-19. Educ Scotl. 2019.

[34] Pagani S, Hunter SC, Lawrence D, Elliott MA (2023). Evaluating mentors in Violence Prevention: a longitudinal, multilevel assessment of outcome change. J Youth Adolesc.

[35] Office H (2023). Violence reduction units, year ending March 2022 evaluation report.

[36] Office H (2018). Serious violence strategy.

[37] Slaby R, Wilson-Brewer R, DeVos H. Final report for aggressors, victims, and bystanders project. Newton, MA: Education Development Center; 1994.

[38] Nebraska Consortium., Bystander Intervention Survey, 10 November 2014. [Online]. Available: http://www.nebraskaconsortium.org/documents/resources/bystander-intervention-survey-behavior-11-10-14.docx. [Accessed 8 Mar 2021].

[39] Galambos NL, Petersen AC, Richards M, Gitelson IB (1985). The attitudes towards Women Scale for adolescents (AWSA): a study of reliability and validity. Sex Roles.

[40] Project MVP. Description of measures: cohort-wide student survey, Available from the Centers for Disease Control and Prevention, National Center for Injury Prevention and Control, Atlanta, GA (unpublished), 2004.

[41] Williams KR, Guerra NG (2007). Prevalence and predictors of internet bullying. J Adolesc Health.

[42] Salmivalli C, Ojanen T, Haanpaa J, Peets K (2005). I’m OK but you’re not and other peer relational schemas: explaining individual differences in children’s social goals. Dev Psychol.

[43] Lereya ST, Humphrey N, Patalay P, Wolpert M, Bohnke JR, Macdougall A, Deighton J. The student resilience survey: psychometric validation and associations with mental health. Child Adolesc Psychiatry Mental Health. 2016;10:44. 10.1186/s13034-016-0132-5 PMC5093941 27822304

[44] Braun V, Clarke V (2013). Successful qualitative research: a practical guide for beginners.

[45] Cissner AB (2009). Evaluating the Mentors in Violence Prevention Program.

[46] Pagani S, Hunter SC, Elliott MA (2022). Evaluating the Mentors in Violence Prevention Program: A process examination of how implementation can affect gender-based violence outcomes. J Interpers Violence.

[47] Kettery HH, Marx RA (2019). Does the gendered approach of bystander programs matter in the prevention of sexual assault among adolescents and college students? A systematic review and meta-analysis. Arch Sex Behav.

[48] Miller E (2018). Reclaiming gender and power in sexual violence prevention in adolescence. Violence against Women.

[49] López DP, López-Nicolás R, López-López R, Puente-López E, Ruiz-Hernández JA (2022). Association between attitudes toward violence and violent behavior in the school context: a systematic review and correlational meta-analysis. Int J Clin Health Psychol.

[50] Carlson J, Casey E, Edleson JL, Tolman RM, Walsh TB, Kimball E (2015). Strategies to engage men and boys in violence prevention: a global organizational perspective. Violence against Women.

[51] Marine S, Trebisacci A (2018). Constructing identity: campus sexual violence activists’ perspectives on race, gender, and social justice. J Coll Student Dev.

[52] Fabiano PM, Perkins HW, Berkowitz A, Linkenbach J, Stark C (2003). Engaging men as social justice allies in ending violence against women: evidence for a social norms approach. Joournal Am Coll Health.

[53] Berkowitz AD (2003). Applications of social norms theory to other health and social justice issues. The social norms approach to preventing school and college age substance abuse: a handbook for educators, counselors, and clinicians.

[54] Mackie G, Moneti F, Denny E, Shakya H. What are social norms? How are they measured? University of California at San Diego - UNICEF Working Paper, San Diego, 2012.

[55] Nolan JM, Schultz PW, Cialdini RB, Goldstein NJ, Griskevicius V (2008). Normative social influence is underdetected. Pers Soc Psychol Bull.

[56] Butler N, Bates R, Quigg Z. Evaluation of the mentors in violence prevention (MVP) programme in Merseyside - Final report. Public Health Institute, LJMU, Liverpool; 2021.

[57] Mellanby AR, Rees JB, Tripp JH (2000). Peer-led and adult-led school health education: a critical review of available comparative research. Health Educ Research: Theory Pract.

[58] Butler N, Quigg Z, Bates R, Jones L, Ashworth E, Gowland S, Jones M (2022). The contributing role of family, school, and peer supportive relationships in protecting the mental wellbeing of children and adolescents. School Mental Health.

[59] Sterrett EM, Jones DJ, McKee LG, Kincaid C (2011). Supportive non-parental adults and adolescent psychosocial functioning: using social support as a theoretical framework. Am J Community Psychol.

[60] Walsh C, Roche E, Gill K. Street doctors Northern Ireland: a mixed-method process and impact evaluation of a youth violence reduction intervention. Evaluation Program Planning. 2023;100. 10.1016/j.evalprogplan.2023.102345 37413885

[61] Panejh S, Nordahl-Hansen A, Cogo-Moreira H (2023). Moving forwards to a world beyond 0.2, 0.5, and 0.8 effect sizes: new cutoffs for school-based anti-bullying interventions. J Interpers Violence.

