# Supplementary material for: The Mentors in Violence Prevention programme: impact on students’ knowledge and attitudes related to violence, prejudice, and abuse, and willingness to intervene as a bystander in secondary schools in England
Source: BMC Public Health. 2024 Mar 6;24:729. doi: 10.1186/s12889-024-18210-9 (PMC10918972; doi:10.1186/s12889-024-18210-9)
Supplement: Supplementary file 1 — Supplementary Material 1 [file 12889_2024_18210_MOESM1_ESM.docx]

**Figure A1: MVP programme logic model**

**Figure A2: MVP programme implementation process model**

**Table A1: MVP programme content**

| Core modules | Focus on the programme core components exploring violence through a gendered lens; developing leadership skills; taking an active bystander approach; exploring the scope of violent behaviour; and, challenging victim blaming |
| --- | --- |
| Additional topics | Online abuse, being left out, rumours, dating abuse, controlling behaviour, sexting, sexual harassment in school, shaming/labelling, carrying weapons, homophobic bullying, transphobic bullying, viewing pornography, child sexual exploitation, impact of pornography on relationships, alcohol and consent, county lines, suicide, racism, disability, insults  Whilst the topic of the social scenario may vary in each session, the mode of delivery and core messages of MVP are the same. |
