# Supplementary material for: The Mentors in Violence Prevention programme: impact on students’ knowledge and attitudes related to violence, prejudice, and abuse, and willingness to intervene as a bystander in secondary schools in England
Source: BMC Public Health. 2024 Mar 6;24:729. doi: 10.1186/s12889-024-18210-9 (PMC10918972; doi:10.1186/s12889-024-18210-9)
Supplement: Supplementary file 2 — Supplementary Material 2 [file 12889_2024_18210_MOESM2_ESM.docx]

**Table S1: Changes in attitudes towards efficacy of violence prevention, mentors and mentees**

|  | Mentors | | | | | Mentees | | | | |
| --- | --- | --- | --- | --- | --- | --- | --- | --- | --- | --- |
| Item | **n** | **Time 1**  **Mean**  **(SD)** | **Time 2**  **Mean**  **(SD)** | **t** | **p** | **n** | **Time 1**  **Mean (SD)** | **Time 2**  **Mean (SD)** | **t** | **p** |
| People’s violent behaviour can be prevented | 142 | 4.10 (0.727) | 4.31 (0.801) | -2.667 | <0.01 | 79 | 3.97 (0.733) | 3.86 (1.034) | 0.853 | NS |
| There are certain things a person can do to help prevent violence | 142 | 4.23 (0.515) | 4.39 (0.770) | -2.231 | <0.05 | 78 | 4.13 (0.727) | 3.94 (0.931) | 1.686 | NS |
| I myself can make a difference in helping to prevent violence | 142 | 3.78 (0.685) | 4.17 (0.781) | -5.662 | <0.001^ | 76 | 3.67 (0.870) | 3.37 (1.018) | 2.382 | <0.05 |
| Doing or saying certain kinds of things can work to help prevent violence | 141 | 4.06 (0.630) | 4.22 (0.775) | -2.234 | <0.05 | 76 | 3.79 (0.838) | 3.70 (1.007) | 0.724 | NS |
| I can learn to do or say the kinds of things that help prevent violence | 140 | 4.22 (0.524) | 4.32 (0.771) | -1.573 | NS | 72 | 3.90 (0.790) | 3.51 (1.007) | 3.050 | <0.01 |
| People can learn to become someone who helps others to avoid violence | 141 | 4.21 (0.571) | 4.33 (0.797) | -1.643 | NS | 74 | 3.99 (0.712) | 3.82 (1.038) | 1.349 | NS |
| Even people who are not involved in a fight can do things that help prevent violence | 141 | 4.14 (0.723) | 4.24 (0.844) | -1.303 | NS | 74 | 3.95 (0.826) | 3.65 (1.039) | 1.935 | NS |
| Even when I’m not involved and it’s not about me, I can make a difference in helping to prevent violence | 140 | 4.01 (0.646) | 4.26 (0.860) | -2.997 | <0.01 | 75 | 3.95 (1.012) | 3.53 (1.143) | 2.448 | <0.05 |

**Note.** Higher scores indicate higher levels of agreement with each statement. SD, standard deviation. ^Statistically significant when controlling for multiple testing (Bonferroni correction).

**Table S2: Changes in bystander attitudes, mentors and mentees**

|  | Mentors | | | | | Mentees | | | | |
| --- | --- | --- | --- | --- | --- | --- | --- | --- | --- | --- |
| Item | **n** | **Time 1**  **Mean**  **(SD)** | **Time 2**  **Mean**  **(SD)** | **t** | **p** | **n** | **Time 1**  **Mean (SD)** | **Time 2**  **Mean (SD)** | **t** | **p** |
| I see myself as a leader | 270 | 3.70 (0.782) | 3.96 (0.866) | -5.110 | <0.001^ | 141 | 3.07 (1.005) | 2.91 (1.041) | 1.805 | NS |
| I see myself as a role model to younger students | 271 | 3.80 (0.737) | 3.96 (0.860) | -2.730 | <0.01 | 141 | 3.19 (1.014) | 3.09 (1.045) | 1.335 | NS |
| I think others see me as a role model to younger students | 257 | 3.58 (0.826) | 3.81 (0.880) | -3.986 | <0.001^ | 126 | 2.91 (1.012) | 2.93 (0.887) | -0.173 | NS |
| I need to set an example in my own behaviour for what I expect in others | 271 | 4.13 (0.719) | 4.21 (0.826) | -1.473 | NS | 134 | 3.85 (0.818) | 3.66 (1.090) | 1.955 | NS |
| It is my responsibility to intervene when I notice a problematic situation | 267 | 3.75 (0.897) | 3.93 (0.846) | -2.619 | <0.01 | 129 | 3.29 (1.049) | 3.32 (1.068) | -0.207 | NS |
| There is no need to get involved in problematic situations | 263 | 2.78 (1.006) | 2.70 (1.097) | 1.082 | NS | 130 | 3.19 (1.188) | 2.89 (1.170) | 2.470 | <0.05 |

**Note.** Higher scores indicate higher levels of agreement with each statement. SD, standard deviation. ^Statistically significant when controlling for multiple testing (Bonferroni correction).

**Table S3: Changes in perceptions of other students’ bystander behaviour, mentors and mentees**

|  | Mentors | | | | | Mentees | | | | |
| --- | --- | --- | --- | --- | --- | --- | --- | --- | --- | --- |
| Item  Students in my school would help out if… | **n** | **Time 1**  **Mean**  **(SD)** | **Time 2**  **Mean**  **(SD)** | **t** | **p** | **n** | **Time 1**  **Mean (SD)** | **Time 2**  **Mean (SD)** | **t** | **p** |
| … a student is making fun of another student | 121 | 2.28 (0.721) | 2.72 (0.686) | -5.337 | <0.001^ | 53 | 3.09 (1.290) | 2.81 (0.810) | 1.543 | NS |
| … a student is spreading rumours and lies about another student behind their back | 117 | 2.21 (0.772) | 2.62 (0.828) | -4.867 | <0.001^ | 46 | 2.78 (1.009) | 2.83 (0.926) | -0.256 | NS |
| …a student is telling lies, spreading rumours, teasing or making fun of another student online (e.g. Facebook, Instagram, Snapchat, TikTok, messages, websites) | 110 | 2.37 (0.822) | 2.66 (0.849) | -3.174 | <0.01^ | 41 | 3.05 (1.244) | 2.93 (0.877) | 0.589 | NS |
| … a student or group of students is pushing, shoving, or trying to pick a fight with another student | 118 | 2.34 (0.879) | 2.69 (0.842) | -4.160 | <0.001^ | 45 | 3.07 (1.031) | 3.07 (1.009) | 0.000 | NS |

**Note.** Higher scores indicate higher levels of agreement with each statement. SD, standard deviation. ^Statistically significant when controlling for multiple testing (Bonferroni correction).

**Table S4: Changes in attitudes towards violence, mentors and mentees**

|  | Mentors | | | | | Mentees | | | | |
| --- | --- | --- | --- | --- | --- | --- | --- | --- | --- | --- |
| Item | **n** | **Time 1**  **Mean**  **(SD)** | **Time 2**  **Mean**  **(SD)** | **t** | **p** | **n** | **Time 1**  **Mean (SD)** | **Time 2**  **Mean (SD)** | **t** | **p** |
| If I walk away from a fight, I’d be a coward (chicken) | 124 | 2.72 (1.108) | 2.44 (1.191) | 2.914 | <0.01 | 46 | 2.50 (1.049) | 2.61 (1.201) | -0.598 | NS |
| I don’t need to fight because there are other ways to deal with being mad | 107 | 4.04 (0.643) | 4.32 (0.667) | -4.422 | <0.001^ | 47 | 4.04 (0.690) | 3.94 (0.818) | 0.961 | NS |
| It’s ok to hit someone who hits you first | 129 | 3.67 (1.112) | 3.29 (1.239) | 5.414 | <0.001^ | 52 | 3.58 (1.258) | 3.50 (1.291) | 0.599 | NS |
| If someone teases me, I usually cannot get them to stop unless I hit them | 123 | 2.12 (0.920) | 2.05 (0.848) | 0.904 | NS | 50 | 2.32 (0.891) | 2.30 (1.055) | 0.159 | NS |
| If I really want to, I can usually talk someone out of trying to fight with me | 79 | 3.76 (0.645) | 3.96 (0.629) | -2.697 | <0.01 | 32 | 3.75 (0.718) | 3.56 (0.669) | 1.139 | NS |
| If I refuse to fight, my friends will think I’m afraid | 117 | 2.49 (1.222) | 2.44 (1.235) | 0.486 | NS | 39 | 2.64 (1.135) | 2.54 (1.211) | 0.530 | NS |

**Note.** Higher scores indicate higher levels of agreement with each statement. SD, standard deviation. ^Statistically significant when controlling for multiple testing (Bonferroni correction).

**Table S5: Changes in gender stereotyping, mentors and mentees**

|  | Mentors | | | | | Mentees | | | | |
| --- | --- | --- | --- | --- | --- | --- | --- | --- | --- | --- |
| Item | **n** | **Time 1**  **Mean**  **(SD)** | **Time 2**  **Mean**  **(SD)** | **t** | **p** | **n** | **Time 1**  **Mean (SD)** | **Time 2**  **Mean (SD)** | **t** | **p** |
| Swearing is worse for a girl than a boy | 124 | 1.82 (0.996) | 1.79 (0.957) | 0.391 | NS | 50 | 1.92 (1.027) | 1.80 (1.010) | 0.667 | NS |
| On a date, the boy should be expected to pay all the expenses | 126 | 2.37 (1.191) | 2.17 (1.146) | 2.557 | <0.05 | 55 | 2.45 (1.184) | 2.42 (1.197) | 0.234 | NS |
| On average, girls are as smart as boys | 126 | 3.67 (1.166) | 3.75 (1.237) | -0.760 | NS | 52 | 3.54 (1.335) | 3.31 (1.528) | 1.014 | NS |
| More encouragement in a family should be given to sons than daughters to go to university | 125 | 1.82 (1.122) | 1.79 (1.109) | 0.355 | NS | 50 | 1.72 (0.970) | 1.82 (1.173) | -0.494 | NS |
| It is ok for a girl to want to play rough sports like football | 132 | 4.60 (0.730) | 4.63 (0.692) | -0.391 | NS | 57 | 4.72 (0.491) | 4.58 (0.706) | 1.530 | NS |
| In general, the father should have greater authority than the mother in making family decisions | 128 | 1.71 (0.957) | 1.77 (1.039) | -0.663 | NS | 54 | 1.94 (1.106) | 1.85 (1.089) | 0.683 | NS |
| It is ok for a girl to ask a boy out on a date | 132 | 4.46 (0.766) | 4.56 (0.657) | -1.735 | NS | 56 | 4.64 (0.483) | 4.46 (0.873) | 1.346 | NS |
| It is more important for boys than girls to do well in school | 132 | 1.61 (0.826) | 1.67 (0.880) | -0.807 | NS | 56 | 1.64 (0.819) | 1.80 (1.135) | -1.242 | NS |
| If both the husband and the wife have jobs, the husband should do a share of the housework such as washing dishes and doing the laundry | 131 | 4.34 (0.950) | 4.20 (1.026) | 1.529 | NS | 53 | 4.23 (1.137) | 4.13 (1.225) | 0.459 | NS |
| Boys are better leaders than girls | 130 | 1.86 (0.986) | 1.69 (0.861) | 2.359 | <0.05 | 56 | 1.70 (0.872) | 1.80 (1.166) | -0.903 | NS |
| Girls should be more concerned with becoming good wives and mothers than having a professional or business career | 128 | 1.55 (0.895) | 1.70 (0.942) | -1.950 | NS | 52 | 1.71 (0.936) | 1.87 (1.155) | -1.016 | NS |
| Girls should have the same freedom as boys | 133 | 4.74 (0.599) | 4.65 (0.769) | 1.268 | NS | 56 | 4.79 (0.530) | 4.66 (0.745) | 1.224 | NS |

**Note.** Higher scores indicate higher levels of agreement with each statement. SD, standard deviation. ^Statistically significant when controlling for multiple testing (Bonferroni correction).

**Table S6: Changes in school participation, mentors and mentees**

|  | Mentors | | | | | Mentees | | | | |
| --- | --- | --- | --- | --- | --- | --- | --- | --- | --- | --- |
| Item | **n** | **Time 1**  **Mean**  **(SD)** | **Time 2**  **Mean**  **(SD)** | **t** | **p** | **n** | **Time 1**  **Mean (SD)** | **Time 2**  **Mean (SD)** | **t** | **p** |
| At school, I decide things like class activities or rules | 134 | 2.57 (1.134) | 2.76 (1.203) | -1.836 | NS | 66 | 2.05 (1.208) | 2.12 (1.209) | -0.456 | NS |
| I do things at school that make a difference (i.e. make things better) | 130 | 3.14 (0.994) | 3.31 (1.092) | -1.891 | NS | 62 | 3.08 (1.121) | 2.73 (1.203) | 2.350 | <0.05 |

**Note.** Higher scores indicate higher levels of agreement with each statement. SD, standard deviation. ^Statistically significant when controlling for multiple testing (Bonferroni correction).
